# IDEAL, the Infectious Diseases of East African Livestock project open access database and biobank

**DOI:** 10.1038/s41597-020-0559-7

**Published:** 2020-07-09

**Authors:** Rebecca Callaby, Cezar Pendarovski, Amy Jennings, Samuel Thumbi Mwangi, Ilana Van Wyk, Mary Mbole-Kariuki, Henry Kiara, Philip G. Toye, Steve Kemp, Olivier Hanotte, Jacobus A. W. Coetzer, Ian G. Handel, Mark E. J. Woolhouse, Barend Mark de Clare Bronsvoort

**Affiliations:** 1 The Epidemiology, Economics and Risk Assessment (EERA) Group, The Roslin Institute, Royal (Dick) School of Veterinary Studies, University of Edinburgh, Edinburgh, UK; 2 Centre for Tropical Livestock Genetics and Health (CTLGH), Easter Bush Campus, Midlothian, UK; 3 Paul G. Allen School for Global Animal Health, Washington State University, Washington, USA; 4 Institute of Tropical and Infectious Diseases, University of Nairobi, Nairobi, Kenya; 5 Hans Hoheisen Wildlife Research Station, University of Pretoria, Pretoria, South Africa; 6 African Union Interafrican Bureau for Animal Resources (AU-IBAR), Nairobi, Kenya; 7 International Livestock Research Institute (ILRI), Nairobi, Kenya; 8 Centre for Tropical Livestock Genetics and Health (CTLGH), ILRI Kenya, P.O. Box 30709, Nairobi, Kenya; 9 Centre for Tropical Livestock Genetics and Health (CTLGH), ILRI Ethiopia, P.O. Box 5689, Addis Ababa, Ethiopia; 10 Cells, Organisms and Molecular Genetics, School of Life Sciences, University of Nottingham, Nottingham, UK; 11 Department of Veterinary Tropical Diseases, Faculty of Veterinary Science, University of Pretoria, Pretoria, South Africa; 12 Usher Institute, Deanery of Molecular, Genetic and Population Health Sciences, University of Edinburgh, Edinburgh, UK

**Keywords:** Agriculture, Genetics, Ecology, Infectious diseases

## Abstract

The Infectious Diseases of East African Livestock (IDEAL) project was a longitudinal cohort study of calf health which was conducted in Western Kenya between 2007–2010. A total of 548 East African shorthorn zebu calves were recruited at birth and followed at least every 5 weeks during the first year of life. Comprehensive clinical and epidemiological data, blood and tissue samples were collected at every visit. These samples were screened for over 100 different pathogens or infectious exposures, using a range of diagnostic methods. This manuscript describes this comprehensive dataset and bio-repository, and how to access it through a single online site (http://data.ctlgh.org/ideal/). This provides extensive filtering and searching capabilities. These data are useful to illustrate outcomes of multiple infections on health, investigate patterns of morbidity and mortality due to parasite infections, and to study genotypic determinants of immunity and disease.

## Abstract (summary)

**Table:** 

Measurement(s)	Health • disease • genotype • Phenotypic variability
Technology Type(s)	Examination • single-nucleotide polymorphism analysis
Sample Characteristic - Organism	Bos indicus
Sample Characteristic - Environment	farm
Sample Characteristic - Location	Kenya

Machine-accessible metadata file describing the reported data: 10.6084/m9.figshare.12459737

## Background & Summary

Animal health research has traditionally focused on specific infections. However, livestock in the tropics are routinely exposed to a wide variety of pathogens whose direct and indirect impacts on animal health are unlikely to be independent of one another^1^. Local breeds have been reared in these heavy disease challenge settings for many centuries, which has likely resulted in selection for broad disease resistance possibly at the expense of higher production^2^.

The Wellcome Trust funded the Infectious Diseases of East African Livestock (IDEAL) project to investigate the relationship between the host and its pathogens in such a context. This project was a multi-disciplinary study designed to address the underlying lack of baseline epidemiological data about infectious disease in Western Kenya and investigate the concept of a ‘good calf’ by focusing on what combination of a calf’s life history (infectious disease exposure, genetic traits, mothering, husbandry practice, and environmental factors) result in a healthy productive calf, or conversely, a poorly grown calf or dead. In other words, why do some infected individuals suffer very few adverse clinical or productivity impacts, where other infected animals in the same environment are severely affected?^3^.

**Fig. 1:**
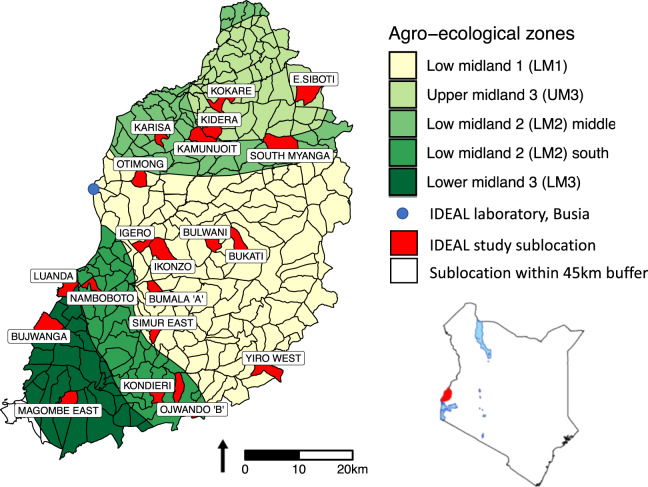
Map of Kenya showing the study area, the 4 agro-ecological zones and sublocations. The sampled sublocations are highlighted. In the LM zones, the annual mean temperature is 21–24 °C, minimum of 14 °C whilst the annual mean temperature in the UM zones is slightly cooler at 18–21 °C (minimum temperature 11–14 °C)^9^. Humidity is highest in zones labelled one, and decreases to semi humid in zones labelled three. Cattle can be found in all zones and the main crops grown in each region is as follows: LM1: sugarcane zone; LM2: marginal sugarcane zone; LM3: cotton zone; UM3: marginal coffee zone^9^. In the study area, LM2 is split into two by LM1.

**Fig. 2:**
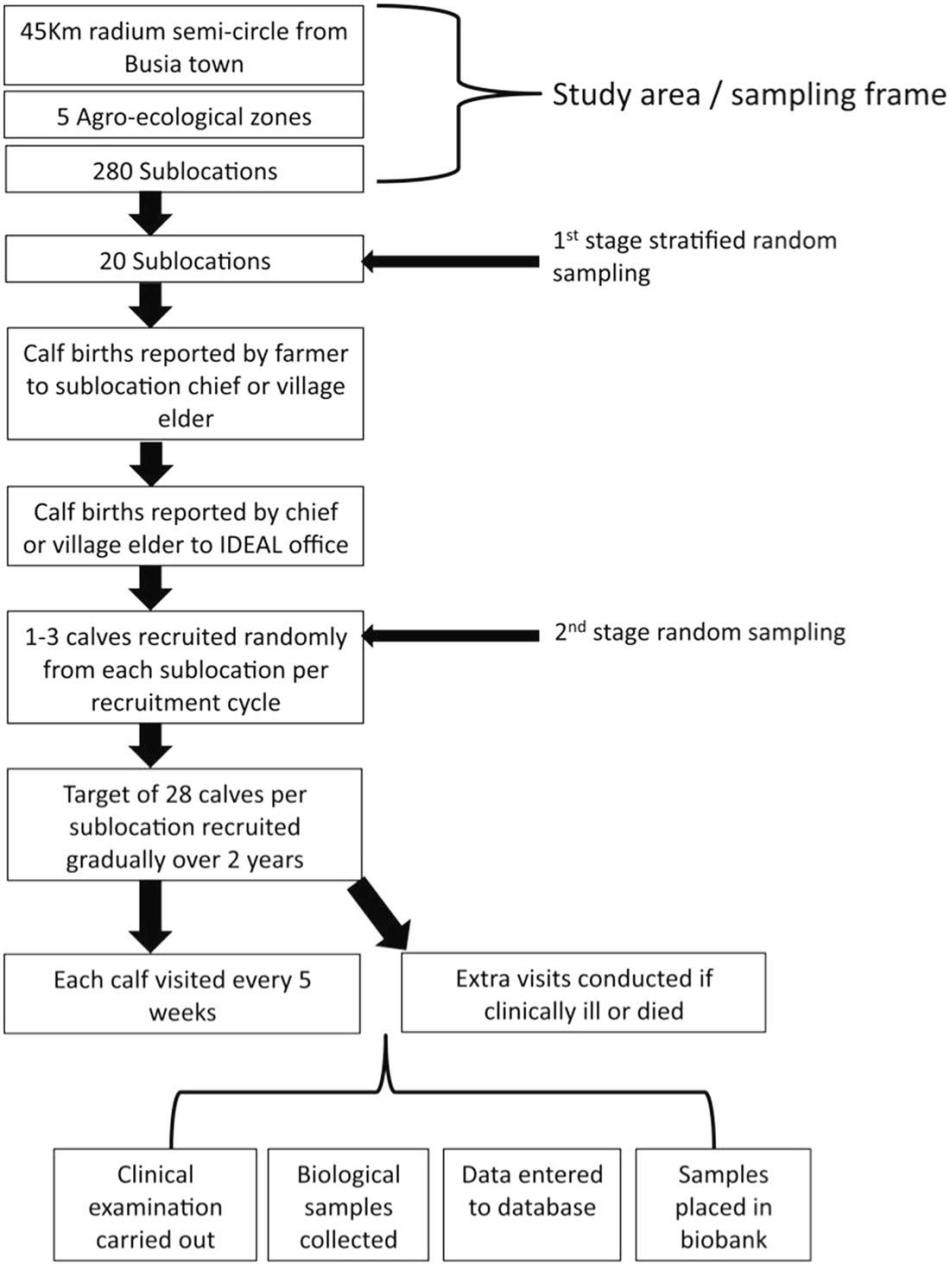
Schema showing the design and sampling used in the IDEAL project. Figure taken from Bronsvoort *et al*.^3^.

**Fig. 3:**
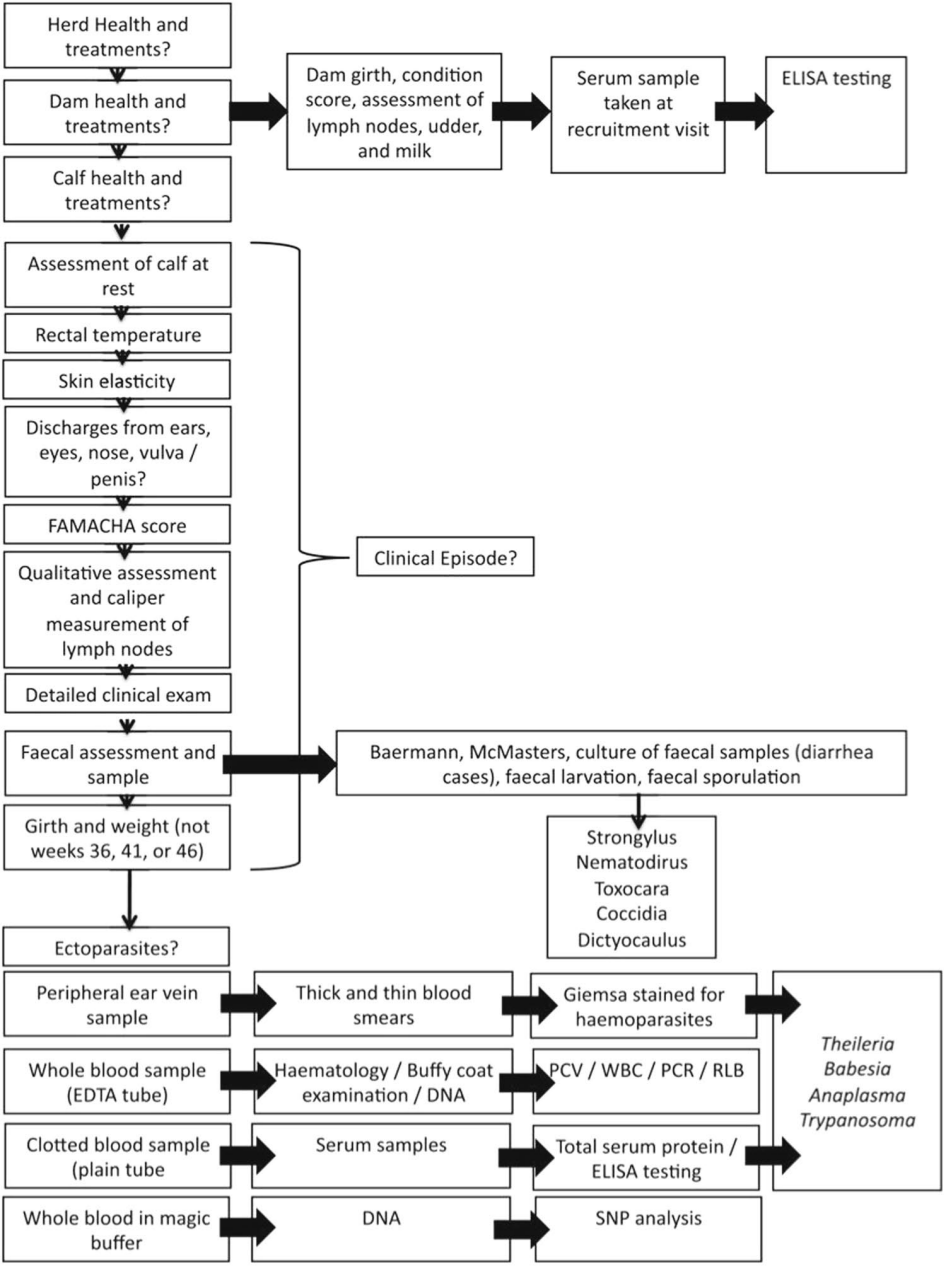
Schema showing the types and timings of clinical examination of calves and the types of sample collection for the IDEAL project. Figure taken from Bronsvoort *et al*.^3^.

Between 2007–2009, 548 new born calves (1–7 days old) from western Kenya (Fig. 1) were recruited to the study. These were recruited from 20 randomly selected sublocations (the smallest administrative unit in Kenya) and followed for the first year (51 weeks) of life. A team of veterinarians and animal health assistants visited each calf every 5 weeks and carried out a clinical examination. Following this examination biological samples were collected. At each visit, whole blood, serum, and faecal samples were collected. Dependent on findings from the clinical examination, appropriate additional diagnostic samples were collected. If the farmer reported that the calf was experiencing clinical disease out with these routine visits, then an interim visit was carried out (a clinical episode visit). If the calf died, then a post-mortem examination was performed, macroscopic findings were recorded contemporaneously, and samples were collected and stored. The clinical, diagnostic, gross and histological post-mortem findings were reviewed and a cause of death was assigned, where possible (Fig. 2). Samples were screened for over 100 different pathogens or infectious exposures, using a range of diagnostic methods (Fig. 3). In addition to pathogen-based tests, the cattle were genotyped using a 50K Illumina^®^ BovineSNP50 BeadChip. Aliquots of remaining sample are stored in a biorepository at the International Livestock Research Institute (ILRI), in Nairobi, Kenya.

The online open-access database presented here, contains all the data collected during the IDEAL project and links to the biorepository of samples held at ILRI in Kenya. Originally the data was stored in a Microsoft^®^ Access field database, which was designed for easy capture of information as it was collected. However, this format was not easily accessible without in-depth knowledge of the study. Here we have simplified the field database reducing it from ~217 tables into a forward-facing open-access research database of 8 tables. These tables are associated with search forms and detailed protocols. This facilitates easy access to the information (see http://data.ctlgh.org/ideal/).

The database is being continuously updated, as new diagnostic tools such as the amplicon and sequence based “haembiome” and “nemabiome” tools generate new high-resolution infection phenotypes from the biobanked material. This allows us to continue to develop our understanding of the differences identified between the calves as well as the impact of infections on growth and immune responses and how these are associated with specific genotypes.

The database can be used with the extensive biobank. This, at the time of publishing, contains over 20,000 serum, DNA and tissue samples. These are available for use in association with the database under certain circumstances.

In terms of a summarising the data contained within the database, 548 calves enrolled into the original study and 5,337 routine visits were carried out. Of the 548 calves, 275 calves were observed to have one or more clinical episodes, and 88 calves died before 51 weeks of age. Therefore, 54% of calves reached one year of age without a reported or observed clinical episode.

**Fig. 4:**
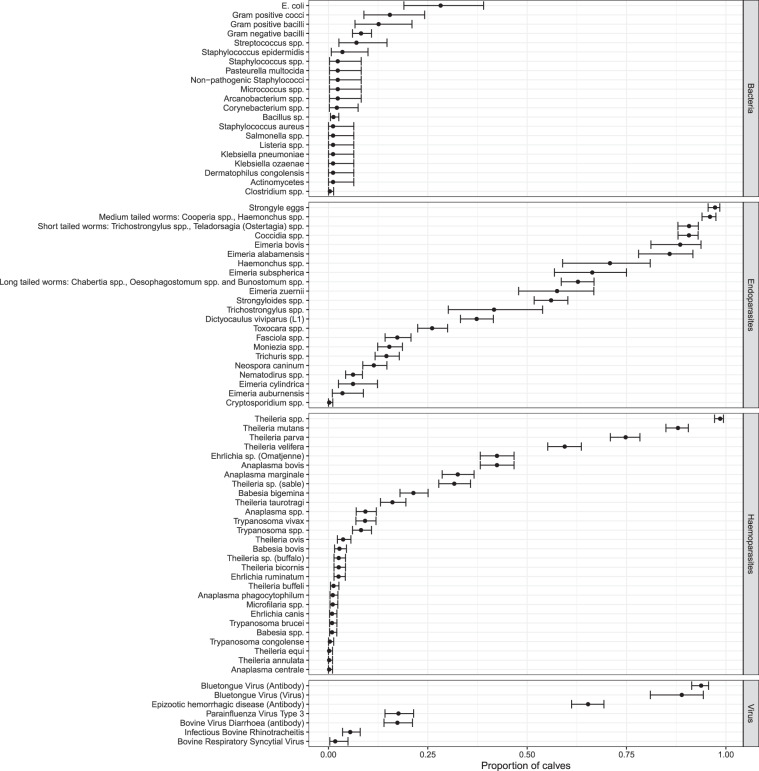
The proportion of calves which tested positive for a given pathogen at any time through the course of the 51 weeks of observation in the IDEAL project. The error bars represent the 95% percent confidence intervals.

**Fig. 5:**
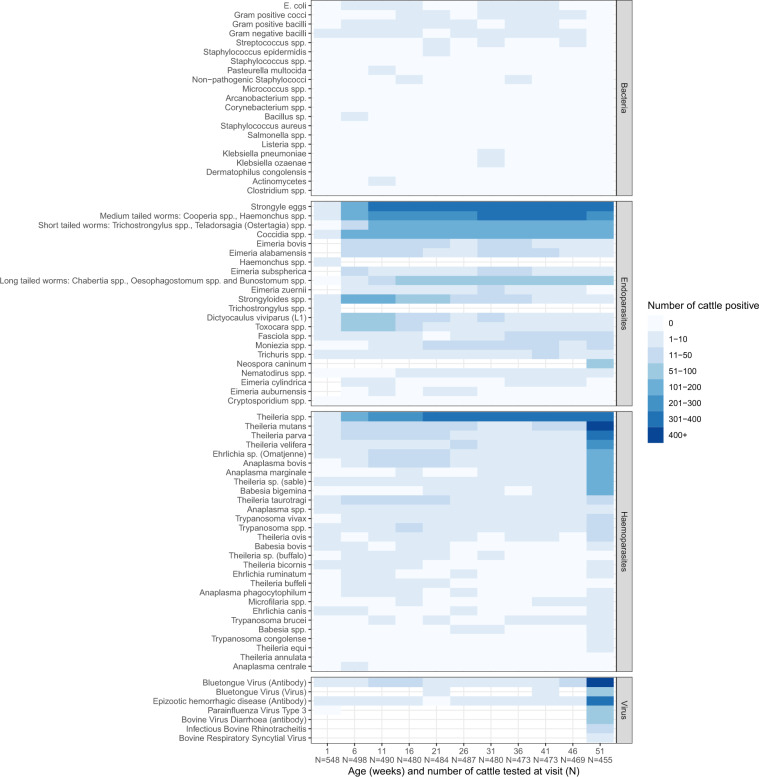
The number of calves which tested positive for a given pathogen at each of the 5 weekly routine visits in the IDEAL project. The total number of calves sampled at each visit is stated below the age on the x-axis. 88 calves died and a few individuals were censured as they received treatment following illness during the course of the study, so by 51 weeks of age only 455 of the original 548 calves remained in the study.

Nearly all the calves were infected with one or more haemoparasites and/or gastrointestinal parasites (Fig. 4). Whilst, Fig. 5 shows the number of animals positive for a given pathogen at each of the 5-weekly routine visits, nearly all cattle were infected with *Theileria* spp. and Strongyle eggs by 51 weeks of age. Mortality was mainly caused by East Coast fever *(T. parva)*, haemonchosis (*Haemonchus spp.)*, and heartwater (*Ehrlichia ruminantium*). The number of parasites is continuously growing as more tests are carried out.

In summary, this evolving dataset linked to its biorepository represents the first attempt to study the complete pathogen landscape of any species and a unique resource for the research community interested in infectious diseases of cattle in East Africa. The longitudinal collection of data allows the outcomes of multiple infections to be related to their effect on their host and their interaction with host genotype. We hope that this database will provide the opportunity for others to study the dynamics of infectious diseases, either as stand-alone work or used in synergy with other projects. The online format integrated with the biobank provides an opportunity to apply any new tools as they become available allowing new questions to be addressed.

## Methods

The design and basic descriptive epidemiology of the IDEAL project is fully described in Bronsvoort *et al*.^3^. To give the IDEAL database a context, we briefly describe the study in relation to the samples taken.

### The study population

Between 2007–2009, 548 free-grazing indigenous East African Shorthorn Zebu calves in Western Kenya were recruited. The project was based in Busia, a town in the west of Kenya on the border with Uganda. The town had a veterinary lab that was able to be used and developed by the project.

Calves were recruited using a stratified two-stage random cluster study design (Fig. 1). In the first stage, a weighted random sample of 20 sublocations was selected from across the four agro-ecological zones represented in the project area. This area was roughly 45 × 90 km^2^. This area was chosen because each area would be drivable from Busia and back to the lab and all visits completed within a day.

In the second stage, approximately 28 3–7 day-old calves were randomly recruited from each sublocation. The sublocations were visited on a rolling 5-week cycle to ensure there was an even distribution of calves recruited across space and season over the study period. Only one calf per dam was recruited and a farmer could only have one calf at a time in the study. Recruited calves were followed for their first year (51 weeks) of life. A calf was selected at random from all those available and eligible on the day of the recruitment visit.

As described in Bronsvoort *et al*.^3^, each calf had to meet the following inclusion criteria to be enrolled in the study: (1) it was between 3 and 7 days old at recruitment; (2) it was not born as a result of artificial insemination of the dam; (3) the dam was not managed under zero-grazing conditions (as this is likely to reflect cattle associated with dairy production and potential exotic genetics and thus not be representative of the traditional small holder farming system); (4) it did not have any congenital deformities (the project was interested in infectious causes of disease and the association with host genetics, rather than direct genetic disease). Recruitment was conditional on the farmer allowing access to the calf, willingness to report clinical episodes to the project team, and agreeing not to self-treat their calves. Farmers were compensated at a rate agreed with the staff of the District Veterinary Office; this comprised the estimated cost of raising the calf for one year as calves were nominally owned by the project for that year. Owners were free to refuse to participate^3^ and consent was obtained in a language in which the participant was confident.

There were two periods during the IDEAL project when sampling and recruitment was suspended. In the first, field work was suspended for 6 weeks in 2008 due to political unrest. This resulted in a small number of calves missing one or two 5 weekly visits. The second was due to an extended holiday period that resulted in staffing problems in 2009/2010.

### Data collection

Below, we briefly describe how the IDEAL data was collected. See Bronsvoort *et al*.^3^ for more details.

### Routine clinical examination of calves

At the recruitment visit, a household questionnaire was completed by interview with the calf owner or head of the household. This questionnaire collected information about the farmer and the farm, such as the type of livestock kept, and animal management practices. Calves were raised according to the farmer’s practices.

Calves received a routine systematic physical examination from a veterinary surgeon at the recruitment visit and these examinations where repeated every 5 weeks until the calf was 51 weeks old. Some routine measurements were taken during the exam (body weight and lymph node width). All abnormalities were noted and all areas were noted as checked. Ectoparasites found were identified. During each visit a standard set of biological samples were taken for further laboratory analysis. In addition, a questionnaire was carried out at each visit to update the IDEAL project about other activities on the farm such as illness or treatment of the other livestock and animal movements. At the final visit, physical phenotype was recorded following a standardised format. The study design, sampling procedure, clinical examinations and sample collection are summarised in Figs. 2 and 3, and can also be read in detail in the supporting documentation online (http://data.ctlgh.org/ideal/).

### Clinical episodes and post-mortem examinations of calves

If a calf became unwell, the farmer was asked to contact the IDEAL project veterinary surgeon. The calf was visited and a full clinical examination and history was taken. Both routine and additional appropriate specific samples were collected based on the syndrome observed^3^. If a calf was deemed to be suffering and if that suffering would be alleviated with treatment, that treatment was given and the calf was no longer visited and was censored from the study from that point. If calves were suffering and that could only be alleviated by euthanasia, this was carried out and the calf was examined post-mortem following the standard protocol. Following the death or euthanasia of a calf, a full gross post-mortem examination was carried out using standard veterinary procedures and clinically appropriate samples collected for further analysis. Cause of death was attributed using a panel of experts with access to all available diagnostic results and the necropsy report. This was carried out for all calves that died at a single timepoint after the close of the project.

### Examination of the dams

A limited clinical examination of the dam was performed at recruitment and in subsequent routine visits until the calf was weaned. At these visits the girth was measured and body condition was scored and the udder was examined for evidence of lesion or mastitis that could affect calf nutritional intake. Phenotypic measurements of coat colour and pattern, horn length and shape, ear shape, size of hump and dewlap was recorded at recruitment^3^.

### Laboratory analysis

Blood samples collected into EDTA tubes were used for differential blood cell counts, performed using the pocH-100iV Diff (Sysmex^®^, Europe GMBH). The haematological parameters investigated are listed in Online-only Table 1. In addition to the automated blood analysis, EDTA unclotted samples were used to make thin blood smears for manual differential cell counts. These smears were transported to the University of Pretoria, South Africa, where blood smears were stained with Diff Quick (Kyron, South Africa) for differential counts. Packed cell volume (PCV) was measured manually using a Hawksley micro-hematocrit reader^4^. Total serum protein (TSP) was measured from 100 μL serum using a refractometer (model RHC-200ATC, Westover Scientific).

Peripheral ear vein blood smears were collected and examined for haemoparasites by microscopy. Thin smears were fixed using methanol and stained using Giemsa. Thick smears were directly stained. One hundred fields were examined under an oil immersion lens. Haemoparasites present were identified to genus level.

Reverse line blot (RLB) hybridization assay was performed as previously described^5^ to detect tick-borne haemoparasites in the blood (*Theileria*, *Anaplasma*, *Ehrlichia*, and *Babesia* (Online-only Table 1))^5^.

The p104 nested PCR was carried out on calves where ECF was suspected on clinical grounds to specifically identify *T. parva*^6^.

Whole blood collected in EDTA was mixed in sodium EDTA tubes in a 1:1 ratio with ‘magic buffer’ (which acted as an anti-coagulant, anti-fungus, anti-bacterial and preservative; Biogen Diagnostica, Villaviciosa De Odon, Spain) at the recruitment visit in readiness for genomic analysis. DNA was extracted from these samples using the Nucleon Genomic DNA extraction kit (TepnelnLife Sciences, Manchester, UK). The Illumina^®^ BovineSNP50 v. 1 BeadChip (Illumina Inc., San Diego, CA, USA) used to genotype the cattle. Genotyping of the 548 calves was carried out at the USDA-ARS bovine functional (Beltsville, MD, USA) and GeneSeek (https://genomics.neogen.com) laboratories using the genome assembly v3.0. In addition, due to the cost of sequencing at the time, a subset of 114 cattle were genotyped using the Illumina^®^ BovineHD Genotyping BeadChip.

Faecal samples were also collected during the study and these where routinely screened using the standard Baermann and McMasters protocols^7^. The number of strongyle eggs per gram of faeces was evaluated using the McMasters counting technique. These could be read to the nearest 50 eggs per gram. Sedimentation was carried out for detection of fluke eggs and larval cultures were used to speciate strongyle eggs^7^. Species where reported at the highest level; strongyle eggs/strongyloides/coccidia/nematodirus. See Online-only Table 1 for more details on the parasites detected.

Serum samples were collected from blood collected into plain vacutainer tubes. These were stored in duplicate for serological analyses. Species-specific antibody response enzyme-linked immunosorbent assays (ELISAs) were performed and analysed according to the manufacturer’s instructions.

The full list of pathogens and viruses for which the cattle have so far been screened can be found in Online-only Table 1. The prevalence of pathogens currently identified across the whole study period and at each visit is presented in Figs. 4 and 5.

Since the database is linked to a biobank, the list of pathogens tested and screened for is continuously updated as new tests are performed or new tools are developed.

## Data Records

The original database was designed to be continuously auditable, with several aims in mind: to capture data from the field and laboratory teams; to allow the management team to check on daily processes; to allow early identification of problems; and to ensure information integrity throughout the database and biobank. This resulted in an extremely complicated structure that required a working knowledge of the project logistics to navigate. For ‘public’ use, the database has been simplified, collecting the data into logical dataframes relating to data about (1) the calves; (2) the farm; (3) the dams; (4) the test results; (5) post-mortem results; (6) clinical illnesses; (7) samples collected and (8) a follow-up study (see below).

Biological samples taken during the project were biobanked at the International Livestock Research Institute (ILRI) Nairobi, Kenya where they continue to be maintained and made available for the wider scientific community.

### The New IDEAL database structure

The new simplified IDEAL database is now accessed through a web application, hosted at http://data.ctlgh.org/ideal/ at the time of publication. It contains all the phenotypic and genetic data, meta-data and study protocols associated with the IDEAL project. It also provides a stand-alone description of the IDEAL project and the data collected.

**Fig. 6:**
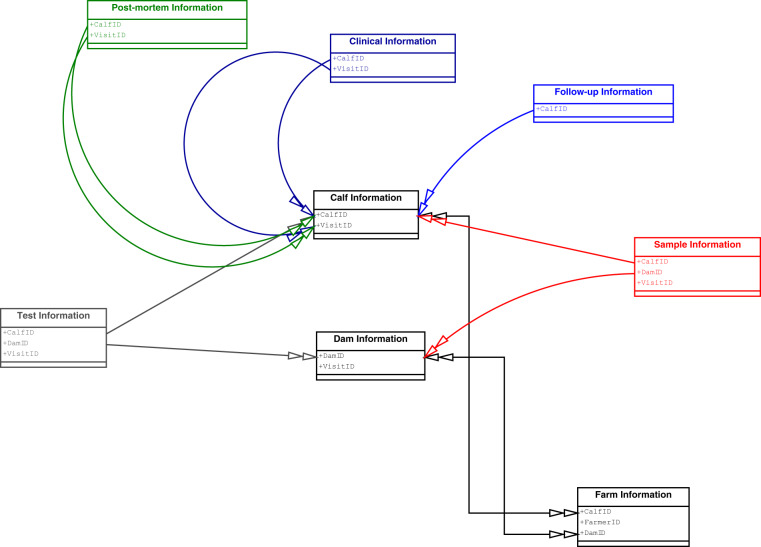
Schema displaying the relations between the 8 views in the application. The bidirectional relations have arrows on their both ends, whereas the unidirectional relations have one arrow pointing to the target view and are of same colour as the source view. Only the IDs for farm, calf, dam and visit are shown. All other fields are omitted due to their large number for the sake of readability.

The database itself consists of 8 tables containing all the data collected during the IDEAL project (Fig. 6). Each database table is mapped to a model entity in the web application with its contents displayed on a separate web page. There are additional web pages which can be accessed through the homepage which provide access to the genetic and meta-data collected during the study. A more detailed description of the information contained within each table and the web application is provided below:

1. **Farm information**: Contains all of the farm level information which was collected at the recruitment visit using the main household questionnaire. This includes details about the farms, type of animals kept and animal management practices. There is only one row per calf in this table as only one calf was recruited per farm, so in total this table consists of 548 rows. To comply with the General Data Protection Regulation (EU GDPR), all the information collected about the farmer during the main household questionnaire has been removed from the online publicly available data.
2. **Calf information**: Contains all the information collected about the calves at each visit. This includes assessments of the calves health, weight and girth measurements, rectal temperature, presence of ectoparasites. In addition it has some information about the wider herd (for example movements in or out and incidences of illness or treatment in the rest of the herd). There is one row per visit in this table, as each calf received multiple visits. This means that static phenotypic information such as coat colour and horn profiles is repeated for each calf across these visits. Therefore, there are 5641 rows in this table, consisting of 5337 routine visits, 216 clinical visits and 88 post-mortem visits for the 548 calves.
3. **Dam information**: Contains all the information about the dams at each visit. This includes subjective assessments of the dam’s health, body condition score, girth measurements, ‘California Mastitis Test’ results and disorders of the udder areas. As with the calf data, there is one row per visit in this table, as each dam received multiple visits until the calf was weaned. There are 5044 rows in this table. Static phenotypic information such as coat colour and horn profiles is repeated for each dam across these visits.
4. **Test information**: Contains results of all the diagnostic tests carried out on the blood and faecal samples. Since multiple diagnostic tests were carried out on dams and calves at each visit, there are multiple rows per visit per dam or calf. This table will be updated as more tests are carried out on the biobanked samples. At the time of publication there are 504865 rows. A list of all the tests which have been carried out on the IDEAL samples so far is given in Online-only Table 1.
5. **Clinical information**: Contains details about all clinical episodes the calves experienced. This table includes information on the body part affected by the disorder, the disorder/lesion and the extent of the lesion. There are 361898 rows as each lesion/disorder and body part is included as a separate row in the table.
6. **Post-mortem information**: Contains information on the cause of death for each of the 88 calves which died as gathered by the post-mortem reports. There is one row per calf in this table.
7. **Sample information**: Links the samples taken at each visit to the samples stored in the biobank. There are 60694 rows in this dataset, as multiple samples were taken at each visit and some of these sample were decanted to make daughter samples.
8. **Follow-up study**: A year after the final visit was made to the IDEAL cattle, a follow-up questionnaire was carried out asking about the status of the calf and whether or not it had offspring. This follow-up information is contained within this table. There are 548 rows in this table.
9. **Genetic information**: This is a web page, which contains the Illumina^®^ BovineSNP50 BeadChip v. 1 and the Illumina^®^ BovineHD Genotyping BeadChip genotypes for the IDEAL cattle. The genetic information can be downloaded and linked backed to the phenotype information using the calf’s unique identity code.
10. **Meta-data**: The meta-data, including the original protocols and standard operating procedures, can be found on the ‘about’ page of the IDEAL web application. The ‘about’ page also includes a list of all the publications which stem directly from the IDEAL project http://data.ctlgh.org/ideal/documentation/about/.

The application itself contains a data descriptor for each table, which provides some background to the table. It also contains links to the associated meta-data and a data dictionary with definitions of every column included in the table and its units of measurement if relevant. See the usage notes below for information on the IDEAL web application.

It is possible for the user to query the database using the search toggle and download delimiter-separated values (DSV) files of variables as required. The test information table may change between downloads as new tests are carried out on the biobanked samples. For example, data on serum vitamin D levels and novel sequence-based identification of haemoparasites will soon be added.

The biobank originally consisted of samples contains 6,692 whole blood samples with two additional aliquots of each stored in cryotubes when the field study finished. See Online-only Table 2 for more details of samples taken. Since these samples are a limited and depletable resource, the numbers of samples available will decrease as additional tests are carried out. The remaining samples are available upon request, dependent on conditions being met. These conditions are described on the IDEAL website http://data.ctlgh.org/ideal/.

### Stable snapshot stored on Edinburgh DataShare

A stable copy of the IDEAL database in the form of raw CSV files corresponding to each of the tables described above is stored on the Edinburgh DataShare platform^8^. This ensures the long-term storage of the IDEAL project data and allows a persistent Digital Object Identifier (DOI) to be attached to the data. As new diagnostic tests are carried out on the biobanked samples, we will update the IDEAL project website and database. Therefore, the data on Edinburgh DataShare represents a stable snapshot of the database at the time of publication of this manuscript.

The tables stored on Edinburgh DataShare follow the same structure as the one described in the data records section above. In addition, the technical validation and methods to link the tables are the same as the one in the usage notes described below.

## Technical Validation

The IDEAL project was a complex multi-disciplinary effort. A veterinary surgeon/senior animal health assistant (AHA) and one or two AHAs went to each animal and followed a set of protocols to collect the data. These standard protocols are included on the IDEAL database website http://data.ctlgh.org/ideal/documentation/about. Data was recorded using palm pilots (Palm OS^®^ running Satellite Forms (SatelliteForms.net)) at each visit and double checked from paper records.

Initially, the project managed data in a set of linked Access databases (Microsoft Corp.). At the end of the field work the field and laboratory databases were merged and moved to a relational MariaDB (free and open source fork of MySQL) database that could be accessed and updated remotely giving all staff access to the data for analysis. Later the data were aggregated and grouped into seven database tables, leaving the initial tables intact. The data from the follow-up study was stored in an additional separate table. A web application written in Python (using the web framework Django) implementing the MTV (Model-Template-View) design pattern, which is a variant of the MVC (Model-View-Controller) design pattern, was developed. The eight database tables were mapped into the model and view layers of the application. A generic HTML (HyperText Markup Language) template serves all views and delivers the data into a uniform tabular format on a separate web page for each view. In the view layer the columns of each HTML table are divided into logical groups. The user has a possibility to customise the output table by collapsing the desired column groups. A custom search form for every view enables querying the data in a user-friendly way.

In terms of samples, all the samples were moved to ILRI Nairobi and appended to the ILRI laboratory information management system for sample management and tracking. Where possible, samples were stored in duplicate and only one of the duplicates moved at a time to reduce the risk of losing complete sample sets. At ILRI duplicates are stored in separate buildings in either −20 °C or −80 °C freezers or in vapour phase in large liquid nitrogen biobank chambers as appropriate.

## Usage Notes

Unique identification codes have been assigned to the data records to facilitate the tracking of data. This unique identification system allows the database user to link the calf to its dam and farmer as well as back to its visit and sample or test result. The identification system does not relate to any other identification system other than that created by IDEAL project.

Each calf-dam-farmer ‘trio’ [i.e. from the same household] recruited into the study is uniquely identified with a 9-digit barcode as in Table 1. Calves are identified with ‘CA’, that corresponds to a verification number = 1. Dams are identified with ‘DM’, that corresponds to a verification number = 2 and Farmers are identified with ‘FR’ with a verification number = 3. The barcode also includes a numeric code for the sublocation and agro-ecological zone (AEZ) of origin. Finally, each of the 548 calf-dam-farmer ‘trios’ is identified with a unique four-digit ID. For example, if the calf/dam/farm 37 was from the UM3 AEZ (AEZ identification number = 01) and Kidera sublocation (sublocation identification number = 02), this information would be indicated in the set of unique identification numbers as follows: CA010210037; DM010220037; FR010230037.

**Table 1:** The unique 9-digit barcode assigned to each calf-dam-farmer ‘trio’ (i.e. from the same household) recruited into the study.

Asset	Agro-ecological zone	Sublocation	Verification number	Unique ID
CA	01 to 05	01 to 20	1	0001 to 0640
DM	01 to 05	01 to 20	2	0001 to 0640
FR	01 to 05	01 to 20	3	0001 to 0640

There are five agro-ecological zone (AEZ) identifiers in this table despite there being four AEZ in the study area (Fig. 1). This is because the agro-ecological zone, LM2 is split into two by LM1 and so we gave the middle and south regions of LM2 a separate identifier.

Routine 5-weekly visits to calves (including visit data from the recruitment visit) are referred to as ‘VRC’ (Visit Routine Calf). The first 5-weekly visit on a calf is the recruitment visit (VRC01). The last 5-weekly visit is referred to as ‘Yearly visit’ (VRC51). Different actions take place at VRC visits depending on whether these are ‘recruitment’, ‘5-weekly’ or ‘yearly’. Dams were visited on a 5-weekly basis till the weaning of the calf. Routine visits on dams are referred to as ‘VRD’ (Visit Routine Dam). The first 5-weekly visit on a dam is the recruitment visit (VRD01). The last 5-weekly visit, which takes place as soon as the farmer gives notice of weaning, is referred to as ‘Final visit’ (VRDnn). This implies that dams will be monitored for different lengths of time depending on the farmer’s management practices. Different actions take place at VRD visits depending on whether these are ‘recruitment’, ‘5-weekly’ or ‘final’. The calf or dam number included in the barcode font of routine visits corresponds to the Unique ID of each ‘farmer-dam-calf trio’, which ranges from 0001 to 0nnn. For example, the unique identification barcode for the recruitment visit of ‘Calf 37’ and the recruitment visit of the dam would read as ‘VRC010037’ and ‘VRD010037’, respectively.

In addition to the VRC / VRD visits, calves were visited outside the frame of routine visits whenever a clinical episode was reported by local animal health assistants (AHAs) or farmers. Clinical visits outside the frame of routine visits are referred to as ‘VCC’ (Clinical Visit on Calf). Unique identification of these visits is best illustrated through the following examples:

- The first clinical visit on CA010210037 is referred to as VCC010037; the second as VCC020037, the third as VCC030037, etc.
- The first clinical visit on CA010310061 is referred to as VCC010061; the second as VCC020061, the third as VCC030061, etc.

If a recruited calf died before the completion of the study, then a post-mortem examination of the dead calf was conducted. This special type of visit is referred to as ‘VPC’ (Visit Post-mortem Calf). Because only one post-mortem visit is ever conducted on each calf, all VPC visits are ‘VPC01’ and include the unique ID of the calf:

- The post-mortem visit on CA010210037 is referred to as VPC010037.
- The post-mortem visit on CA010310061 is referred to as VPC010061, etc.

Navigating through the data is possible in the web application using the identification codes (Fig. 6). For example, using the CalfID from the Farm Information view offers access to the associated data from the Calf Information view, and vice versa.

Two different formats of delimiter-separated values (DSV) files are available for download: TSV (tab-separated values) and comma-separated values (CSV). Both are suitable for custom data analysis with a programming language of choice (R, Python, Perl, etc.). The former offers better human-readable experience and the latter is the preferred choice for programmatic processing. The data for downloading can be narrowed to the requirements of the user by using the search form and collapsing column groups.

## Online-only Tables

**Online-only Table 1:** Diagnostics tests which have been carried out on the IDEAL samples to screen the samples for pathogens and to produce haematological profiles.

Sample Type	Test	Test result
Faecal	Direct Baermann	Dictyocaulus viviparus (L1)
	McMaster	Coccidia spp., Long tailed worms: Chabertia spp., Oesophagostomum spp., Bunostomum spp., Medium tailed worms: Cooperia spp., Haemonchus spp., Moniezia spp., Nematodirus spp., Strongyle eggs, Strongyloides spp., Toxocara spp., Trichuris spp.
	Flotation	Coccidia spp., Nematodirus spp., Strongyle eggs, Strongyloides spp., Toxocara spp.
	Sedimentation	Coccidia spp., Fasciola spp., Strongyle eggs, Toxocara spp.
	Sporulation	Eimeria alabamensis, Eimeria auburnensis, Eimeria bovis, Eimeria cylindrica, Eimeria subspherica, Eimeria zuernii
	Guildhays Test	Rotavirus
	Faecal culture and larvae identification	Long tailed worms: Chabertia spp., Oesophagostomum spp. and Bunostomum spp., Medium tailed worms: Cooperia spp., Haemonchus spp., Nematodirus spp., Short tailed worms: Trichostrongylus spp., Teladorsagia (Ostertagia) spp.
Peripheral ear vein	Giemsa stain	Anaplasma spp., Babesia spp., Ehrlichia ruminatum, Theileria spp., Trypanosoma spp.
	Blood smears stained with Diff Quick (Kyron, South Africa)	Absolute eosinophil count, Absolute lymphocyte count, Absolute monocyte count, Absolute neutrophil count
	Dark ground microscopy	Microfilaria spp., Trypanosoma spp.
EDTA tube	pocH-100iV Diff (Sysmex^®^ Europe GmbH)	Absolute eosinophil count, Absolute lymphocyte count, Haematocrit, Hemoglobin concentration, Mean cell haemoglobin, Mean corpuscular hemoglobin concentration, Mean platelet volume, Platelet count, Platelet distribution width, Platelet larger cell ratio, Red cell count, Red cell distribution width - coefficient of variation, Red cell distribution width - standard deviation, Total white cell count
	Hawksley micro-hematocrit reader	Packed cell volume
	Reverse Line Blot (RLB)	Anaplasma bovis, Anaplasma centrale, Anaplasma marginale, Anaplasma phagocytophilum, Babesia bicornis, Babesia bigemina, Babesia bovis, Babesia caballi, Babesia canis, Babesia catch-all 1, Babesia catch-all 2, Babesia divergens, Babesia felis, Babesia gibsoni, Babesia gibsoni Japan, Babesia gibsoni USA, Babesia leo, Babesia major, Babesia microti, Babesia occultans, Babesia rossi, Babesia sp. (sable), Babesia venatorum, Babesia vogeli, Ehrlichia / Anaplasma catch-all, Ehrlichia canis, Ehrlichia chaffeensis, Ehrlichia ruminatum, Ehrlichia sp. (Omatjenne), Theileria / Babesia catch-all, Theileria annae, Theileria annulata, Theileria bicornis, Theileria buffeli, Theileria catch-all, Theileria equi, Theileria lestoquardi, Theileria mutans, Theileria ovis, Theileria parva, Theileria separata, Theileria sp. (buffalo), Theileria sp. (kudu), Theileria sp. (sable), Theileria taurotragi, Theileria velifera
	PCR	Bluetongue Virus, Capripox, Ehrlichia ruminatum, Epizootic hemorrhagic disease, Trypanosoma brucei, Trypanosoma congolense, Trypanosoma vivax, p104 for Theileria parva
	Ziehl Neelsen staining for microscopy	Cryptosporidium spp.
Plain tube	Refractometer (model RHC-200ATC, Westover Scientific)	Total serum proteins
	ELISA	Anaplasma marginale, Babesia bigemina, Bluetongue Virus, Bovine Leukaemia Virus, Bovine Respiratory Syncytial Virus, Bovine Virus Diarrhoea, Bovine Virus Diarrhoea (antigen), Epizootic Haemorrhagic Disease Virus, Infectious Bovine Rhinotracheitis, Leptospira hardjo, Neospora Caninum, Parainfluenza 3 Virus, Theileria mutans, Theileria parva, ILG-10, TgF
Ecotoparasites	Identification of ticks	Amblyomma variegatum, Riphicephalus appendiculatus
Bacteriology	Bacteriology conclusion	Actinomyces sp., Actinomycetes, Arcanobacterium spp., Bacillus sp., Clostridium spp., Corynebacterium spp., Dermatophilus congolensis, E. coli, Gram negative bacilli, Gram positive bacilli, Gram positive cocci, Klebsiella ozaenae, Klebsiella pneumoniae, Listeria spp., Micrococcus spp., Non-pathogenic Staphylococci, Pasteurella multocida, Salmonella spp., Staphylococcus aureus, Staphylococcus epidermidis, Staphylococcus spp., Streptococcus spp., Thin rods (Bacteria), Unidentified colony, Weksella zoohelcum

**Online-only Table 2:** The samples collected from the IDEAL cattle and their uses.

Sample Label	Sample Description	Uses	Animal Sampled	Sample Type
BCI	Bacterial Isolates from Swabs	Diagnostics	Calves	Clinical
BFI	Bacterial Isolates from Faeces	Diagnostics	Calves	Clinical
BGA	Aliquot of heparinised blood in 1.5 ml eppendorf tube	Diagnostics	Calves	Routine
BGB	Aliquot of heparinised blood in 1.5 ml eppendorf tube	Diagnostics	Calves	Routine
BGC	Aliquot of heparinised blood in 1.5 ml eppendorf tube		Calves	Routine
BKS	Jugular blood thick smear. Several drops of blood from 5 ml EDTA plastic tubes was used to generate two thick smears of non-peripheral (jugular) blood. A separate slide is used for each smear and they where stored without further processing (unfixed & unstained)	Diagnostics	Calves & dams	Routine
BMI	Bacterial Isolates from milk from California Mastitis Test (CMT) positive quarters	Diagnostics	Dam	Clinical
BNS	Jugular blood thin smear. Several drops of blood from 5 ml EDTA plastic tubes was used to generate two thin smears of non-peripheral (jugular) blood. A separate slide is used for each smear and they were fixed with absolute methanol for 5 minutes before storing	Diagnostics	Calves & dams	Routine
CMS	‘Clinical Miscellaneous Samples’; a heterogeneous group of samples whose collection is ‘in addition’ to the compulsory routine samples	Clinical diagnosis	Calves	Clinical
HSV	1.8 ml plastic cryotubes of jugular blood decanted from 10 ml heparinised glass tubes	Diagnostics	Calves	Clinical
MBE	Mix of jugular blood and magic buffer (5 ml: 5 ml) in 10 ml EDTA glass tubes	Host genetics	Calves & dams	Routine
MKS	Thick smear from peripheral blood collected from the marginal ear vein	Screening of blood borne protozoa	Calves	Routine
MLK	40–50 ml plastic tubes with white lead containing 40–50 ml of milk pulled from all healthy quarters (i.e. quarters presenting with no macroscopic signs of mastitis)	Nutrition studies	Dams	Routine
MNS	Thin smear from peripheral blood collected from the marginal ear vein	Manual haematology & screening of blood borne protozoa	Calves	Routine
NMB	Jugular blood thin smear for new methylene blue staining and reticulocite count	Diagnostics	Calves	Clinical
RED	Jugular blood in 5 ml EDTA plastic tubes	Automated haematology, manual PCV, screening of trypanosomes, PCR	Calves & dams	Routine
RLE	Mix of EDTA jugular blood and RNALater (500 µl: 1.3 ml) in 1.8 ml plastic cryotubes with external thread	RNA studies	Calves	Routine
SER	1.8 ml plastic cryotubes containing serum derived from centrifugation of jugular blood in 10 ml glass-made plain vacutainers	Serology screening	Calves & dams	Routine & clinical
